# Food and social connectedness for young people: an opportunity for public health

**DOI:** 10.1177/17579139261440241

**Published:** 2026-04-11

**Authors:** R Taheem, J Sofaer, M Barker, K Woods-Townsend

**Affiliations:** NIHR Southampton Biomedical Research Centre, University Hospital Southampton NHS Foundation Trust, University of Southampton, Southampton SO16 6YD, UK; Southampton City Council, Southampton, UK; Southampton Institute for Arts and Humanities, University of Southampton, Southampton, UK; Department of Archaeology, Faculty of Arts and Humanities, University of Southampton, Southampton, UK; NIHR Southampton Biomedical Research Centre, University Hospital Southampton NHS Foundation Trust, University of Southampton, Southampton, UK; MRC Lifecourse Epidemiology Centre, Faculty of Medicine, University of Southampton, Southampton, UK; NIHR Southampton Biomedical Research Centre, University Hospital Southampton NHS Foundation Trust, University of Southampton, Southampton, UK; School of Healthcare Enterprise and Innovation, Faculty of Medicine, University of Southampton, Southampton, UK

## Abstract

This article is an opinion paper which reflects on the recently published Food Strategy for England and how it frames the food system in the context of social and cultural factors, which is explored in relation to young people. In relation to outlining these issues, the authors draw out the implications for public health practice in local government.

## Background

Childhood obesity is a challenge for local and national governments. It precedes adult obesity which is a leading cause of poor health and has associated socio-economic consequences.^1,2^ Children living in the most disadvantaged areas in England are twice as likely to be living with obesity as those in the least disadvantaged areas.^3^ In response, local authorities have been encouraged to pursue a whole systems approach.^4^ The recent Food Strategy for England creates a timely opportunity to consider the food system drivers of childhood obesity.^5^ Significantly, the Food Strategy provides the chance to consider the role of social and cultural factors as it situates the food system within the context of social connections, heritage and national pride. However, the emphasis on these factors raises the question as to whether young people experience food in this way and how this informs the delivery of the Food Strategy at a local level.

Our work in the UK Research and Innovation (UKRI)-funded Pathways to Health Through Cultures of Neighbourhoods project (*Pathways*) revealed that young people age 13–18 years perceive commercial food entities as important socio-cultural assets that support social connectedness. This poses both challenges and opportunities for informing directions in public health policy as part of a whole systems approach. At a population level, the obesogenic environment, including the food retail environment, is considered a key opportunity for policy action. However, changing the local retail environment without taking into consideration young people’s social connectedness may lead to unanticipated outcomes, as seen in other areas of systems intervention.^6^

**Figure:**
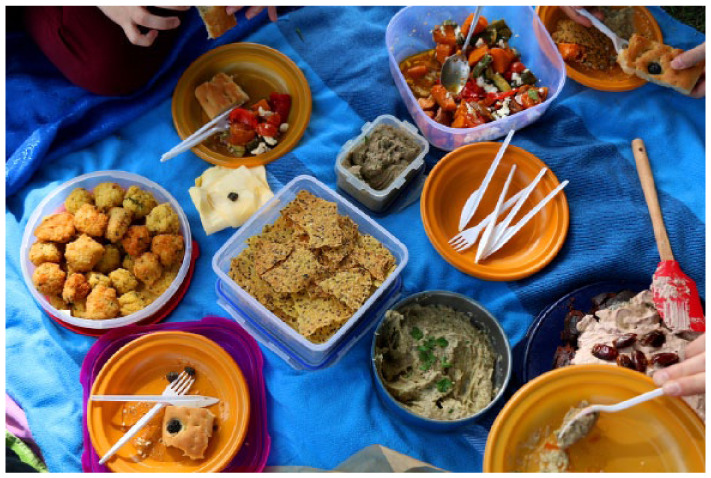


## Food, Social Connectedness and Young People

Commensality (sharing and eating food together) has long been understood in anthropology and archaeology as a multifacetted, cross-cultural and historical nexus for constructing social relations, including community belonging.^7^ These insights have much to offer a whole systems approach to public health, including the materiality of what, where and how foods are consumed.^8^ Critically, food is not a neutral substance but is a cultural phenomenon. Food carries cultural capital, social identities and emotional value; is a venue for symbolic exchange vital to the development of social relations; and is consumed in situations and spatial contexts laden with meaning.^9^

To date, public health research has tended to focus on family and school meals as venues for commensality,^8^ but *Pathways* revealed the role of informal commensality in promoting adolescents’ social connections with peers. In *Pathways*, a diverse group of 174 young people took part in creative workshops and focus groups (creative writing, dance, theatre, photography, mapping) in disadvantaged wards in the east, centre and west of Southampton to understand which places in the city were important to them and what they did there. Across all participants, many of the most important locations were where young people socialised with peers, including eating and drinking. They included supermarkets, fast food outlets, bubble tea cafes, shopping centres and corner shops, all of which were identified by participants as important assets and where commensality was integral to the development of social connectedness.

Given the commercial nature of the primary assets identified by young people, their commensality often centred on the consumption of fast foods, high-sugar drinks and supermarket meal deals. This supports research showing that health is not a priority in adolescents’ values and that food choices outside the home align with peers in order to prioritise ‘relatedness’.^10,11^ The social capital attached to food was also important. For example, consumption of high-sugar bubble teas was considered desirable because it created social capital among young women, and cafes were perceived as safe spaces.

Adolescents are developmentally primed to engage with people outside their families.^12^ Further research is needed, but self-directed commensality among young people may be integral to the development of independent social networks essential for social and emotional development, including a growing sense of autonomy and agency.^13^ It may therefore be an important systems driver underpinning young people’s choices.^14^

As investment in youth clubs and other facilities for young people has been eroded, it is possible that young people are turning to commercial entities because they provide opportunities for in-person social connectedness.

## Implications And Opportunities

A whole systems approach for tackling obesity requires collective understanding that responds to evolving needs of communities and prioritises policies and interventions most likely to change the system and improve long-term outcomes. Neither the current narrative around obesity nor the Food Strategy for England considers the important relationship between young people’s need for social connectedness and the food culture they experience. Similarly, national obesity policy has focussed on individual behaviour change and interventions to improve the food environment through legislation.^15^ However, these too ignore the importance of commensality and relationships to peers as social phenomena influencing young people’s choices.

Understanding the importance of commensality and its role in social connectedness creates opportunities for new policy solutions centring young people’s cultural and social values. Value-led interventions are more likely to be effective for adolescents.^10^ Hence, their values should be considered within systems-based solutions.

Public health policies which typically focus on restricting the concentration of unhealthy outlets may limit what young people consider as ‘assets’. Policies therefore need to consider potential negative impact on young people’s social connectedness. Mitigating negative impact requires multiple local government departments to work together across public health, children’s services and planning to develop strategies and interventions for healthy safe spaces that meet young people’s needs for social connectedness.

## Conclusion

The national focus on the prevention of childhood obesity means that it is crucial to consider the complexity of the relationship between young people and their food experiences.

The National Food Strategy has opened the door to conversations about the role of social and cultural factors in the food system, which we need to consider moving forward. Understanding young people’s values including commensality and social relationships connected with food could help to conceptualise and describe complexities relating to food and health behaviours. This should be an integral part of a ‘whole systems approach’. For their values to be properly understood and articulated, we need to work this through in partnership with young people.
